# Simulated microgravity facilitates stomatal ingression by *Salmonella* in lettuce and suppresses a biocontrol agent

**DOI:** 10.1038/s41598-024-51573-y

**Published:** 2024-01-09

**Authors:** Noah Totsline, Kalmia E. Kniel, Chandran Sabagyanam, Harsh P. Bais

**Affiliations:** 1 Department of Plant and Soil Sciences, University of Delaware, Newark, DE 19713 USA; 2 Delaware Biotechnology Institute, University of Delaware, 311 AP Biopharma, 590 Avenue 1743, Newark, DE 19713 USA; 3 Department of Animal and Food Sciences, University of Delaware, Newark, DE 19713 USA

**Keywords:** Plant physiology, Plant signalling, Plant stress responses, Stomata

## Abstract

As human spaceflight increases in duration, cultivation of crops in spaceflight is crucial to protecting human health under microgravity and elevated oxidative stress. Foodborne pathogens (e.g., *Salmonella enterica*) carried by leafy green vegetables are a significant cause of human disease. Our previous work showed that *Salmonella enterica* serovar Typhimurium suppresses defensive closure of foliar stomata in lettuce (*Lactuca sativa* L.) to ingress interior tissues of leaves. While there are no reported occurrences of foodborne disease in spaceflight to date, known foodborne pathogens persist aboard the International Space Station and space-grown lettuce has been colonized by a diverse microbiome including bacterial genera known to contain human pathogens. Interactions between leafy green vegetables and human bacterial pathogens under microgravity conditions present in spaceflight are unknown. Additionally, stomatal dynamics under microgravity conditions need further elucidation. Here, we employ a slow-rotating 2-D clinostat to simulate microgravity upon in-vitro lettuce plants following a foliar inoculation with *S. enterica* Typhimurium and use confocal microscopy to measure stomatal width in fixed leaf tissue. Our results reveal significant differences in average stomatal aperture width between an unrotated vertical control, plants rotated at 2 revolutions per minute (2 RPM), and 4 RPM, with and without the presence of *S. typhimurium*. Interestingly, we found stomatal aperture width in the presence of *S. typhimurium* to be increased under rotation as compared to unrotated inoculated plants. Using confocal Z-stacking, we observed greater average depth of stomatal ingression by *S. typhimurium* in lettuce under rotation at 4 RPM compared to unrotated and inoculated plants, along with greater in planta populations of *S. typhimurium* in lettuce rotated at 4 RPM using serial dilution plating of homogenized surface sterilized leaves. Given these findings, we tested the ability of the plant growth-promoting rhizobacteria (PGPR) *Bacillus subtilis* strain UD1022 to transiently restrict stomatal apertures of lettuce both alone and co-inoculated with *S. typhimurium* under rotated and unrotated conditions as a means of potentially reducing stomatal ingression by *S. typhimurium* under simulated microgravity. Surprisingly, rotation at 4 RPM strongly inhibited the ability of UD1022 alone to restrict stomatal apertures and attenuated its efficacy as a biocontrol following co-inoculation with *S. typhimurium*. Our results highlight potential spaceflight food safety issues unique to production of crops in microgravity conditions and suggest microgravity may dramatically reduce the ability of PGPRs to restrict stomatal apertures.

## Introduction

Spaceflight production of microgreens offers a promising dietary supplement to ameliorate health risks associated with prolonged exposure to microgravity and cosmic radiation^1,2^. However, the persistence of foodborne pathogens including *Salmonella enterica*, *E. coli*, and *Shigella sonnei* aboard the International Space Station (ISS) presents serious food safety risks^3,4^. Accumulating studies demonstrate foodborne bacterial pathogens utilize virulence factors associated with infection of animal hosts to suppress and evade plant innate immunity for colonization of plant tissues, reflecting a cross-kingdom host range^5–7^. For example, *S. enterica* uses a Type-III Secretion System (T3SS)-dependent mechanism to inhibit the abscisic acid (ABA) biosynthetic pathway in lettuce, along with ABA-regulated stomatal closure^6^. Plants restrict the width of foliar stomata in response to abiotic physiological stresses and as part of innate immunity against pathogenic microbes^8^. While indirect indicators of stomatal dynamics like photosynthesis and evapotranspiration have been measured under microgravity^9,10^, stomatal aperture modulation and foliar physiology under microgravity in general remain unknown. While human bacterial pathogens under microgravity have displayed enhanced virulence in animal hosts, their virulence in plant hosts under microgravity remains unknown^11^. Previous studies have reported increased plant susceptibility to fungal pathogens, potentially attributed to the increased buildup of CO_2_ and ethylene^12^, cell wall loosening and reduced synthesis of lignan^13^, or an uncharacterized molecular mechanism. Under microgravity and simulated microgravity, downregulation of the post-transcriptomic RNA chaperone Hfq is suggested to be the molecular basis for enhanced animal virulence in human pathogenic bacteria^14,15^. Interestingly, Hfq is a regulator of virulence in multiple genera of phytopathogenic bacteria^16^ suggesting its differential expression in cross-kingdom foodborne pathogens under microgravity might influence colonization of plant hosts.

Here, we employed a 2-D clinostat to simulate microgravity through continuous vertical rotation of in vitro plants at 2 and 4 RPM with the longitudinal root to shoot axis perpendicular to the rotation plane. According to the starch-statolith model, plants perceive the gravity vector through sedimentation of starch amyloplasts within statocyte cells present within the shoot endodermis and root columella^17^. As such, plant growth relative to the gravity vector may be dependent on perception of inclination as opposed to intensity of gravitational force^18,19^. 2-D clinostats slowly rotate at 1–4 RPM to simulate microgravity upon plants by maintaining amyloplasts in a constant state of free fall within statocytes, preventing sedimentation within statocytes required for gravitropism^20–24^. Rotation at 2 RPM was sufficient to produce clear disruption of gravitropic root growth compared to unrotated plants in our system (Extended Data Fig. 1). It is important to note that while clinostats negate the directionality of the gravity vector via continuous rotation, they do not provide true microgravity^24^. Additionally, rotation in clinostats can cause mechanical stress via redistribution of weight and bending of tissues^24^. As such, interpreting data provided by clinostat experiments must be viewed in the light of these caveats. Prior to our work, plant interactions with human pathogens under a true or simulated microgravity were unexamined^11^. Plant interactions with bacteria in general are largely unexamined under such conditions, in addition to the ability of pathogens of any class to modulate stomatal width. We hypothesized that the physiological stress of simulated microgravity may enhance constriction of stomatal apertures compared to control plants at both 2 and 4 RPM. We further postulated that *Salmonella* may override closure of stomata from physiological stress and innate immunity for ingression of the apoplast under rotation.

Studies have consistently reported the use of plant-growth promoting rhizobacteria (PGPRs) as biocontrol agents to protect plants against biotic and abiotic stresses^25^. We have previously shown a root-application of the PGPR *Bacillus subtilis* strain UD1022 transiently restricts stomatal apertures in *Arabidopsis*, spinach, and lettuce plants^26,27^, limiting foliar entry of various foodborne bacterial pathogens including *Salmonella*^6,27^. Here, we hypothesized a root-application of UD1022 by itself would restrict stomatal apertures of lettuce under rotated conditions and restrict stomatal apertures when co-inoculated with *S. typhimurium* under rotated conditions.

## Results and discussion

**Figure 1:**
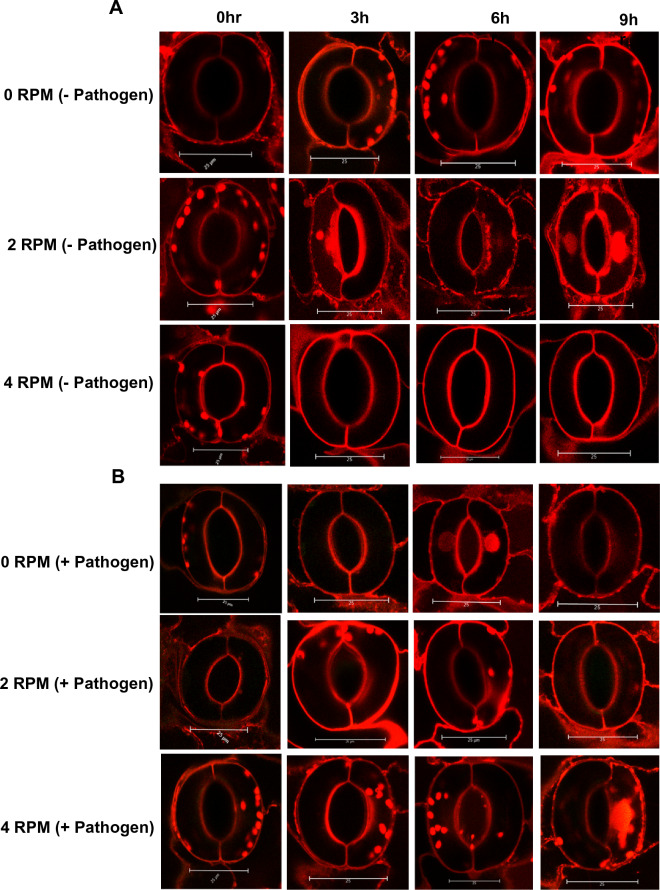
Representative images of stomatal apertures in lettuce plants under simulated microgravity in absence (**A**) and presence of *Salmonella* (**B**). Lettuce plants were subjected to simulated microgravity in a clinostat at 2, and 4 RPM, or a 0 RPM vertical control. Plants were inoculated with *Salmonella enterica* GFP strain. Unrotated plants infected with *S. enterica* were also imaged for stomatal apertures. Average stomatal apertures were micro-graphed and aperture size was estimated from n = 150 stomates per sample.

**Figure 2:**
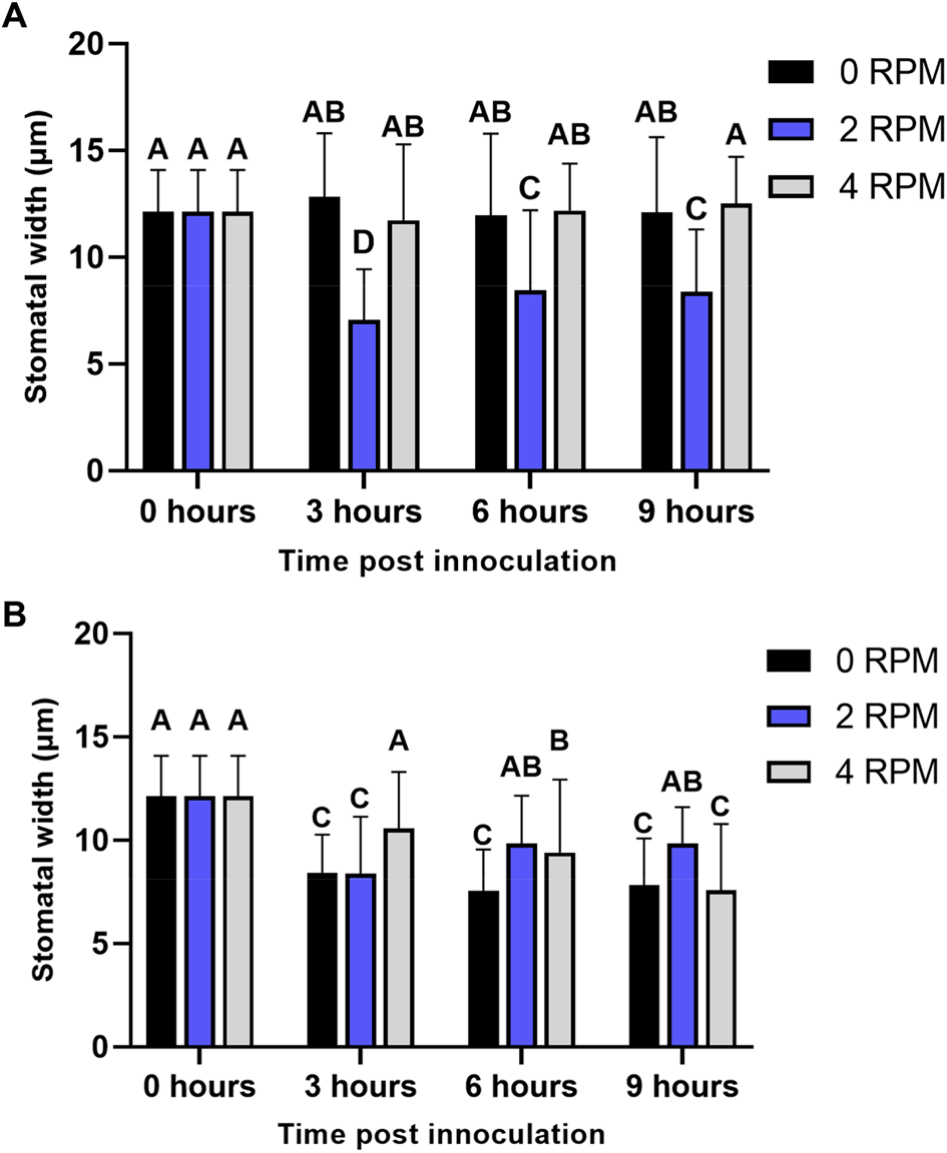
(**A**) Average stomatal aperture width overtime in lettuce plants following mock treatment and maintained vertically as a control (0 RPM) or rotated at 2/4 RPM. (**B**) Average stomatal aperture width overtime in lettuce plants following inoculation with *S. typhimurium* maintained vertically as a control (0 RPM) or rotated at 2/4 RPM. Stomatal apertures were imaged with confocal microscopy and aperture size was measured using ImageJ. At each timepoint, 3 leaves were harvested (one per plant), and 25 stomata were measured from each leaf. This was repeated in two experimental replicates (n = 150 stomata total per timepoint). Letters indicate statistical similarity between timepoints within a treatment determined using an all-pairs Tukey HSD test (p < 0.05).

Here, we simulated microgravity for lettuce plants following either a foliar application of GFP-labeled *Salmonella enterica* serovar Typhimurium 14028 or a mock treatment (uninoculated). We collected leaf tissue for fixation and confocal microscopy at experimental timepoints following rotation for quantification of stomatal widths at 3, 6, and 9 h post inoculation/rotation (Figs. 1A,B, 2; Extended Data Fig. 2A,B, Extended Data Movie 1). Plants rotated without *Salmonella* displayed dramatic differences in average stomatal aperture width between 2 and 4 RPM, with plants rotated at 2 RPM displaying greatly reduced average aperture sizes at 3-, 6- and 9-h post-rotation compared to the same experimental timepoints in uninoculated plants rotated at 4 RPM or unrotated (Fig. 2A,B). Uninoculated plants rotated at 4 RPM showed no statistical differences in average stomatal aperture width at any experimental timepoint compared to uninoculated, unrotated plants (Figs. 1A,B, 2A,B). Lettuce plants inoculated with *Salmonella* and rotated at 2 RPM showed wider average stomatal width at 6- and 9-h post *Salmonella* treatment compared to inoculated plants which were not rotated, while rotation of inoculated plants at 4 RPM displayed wider average stomatal width at 3- and 6-h compared to inoculated and unrotated plants (Figs. 1A,B, 2A,B). The results suggest that simulated microgravity provides *Salmonella* an advantage in stomatal ingression compared to the unrotated plants, highlighting potential food safety concerns for spaceflight production of leafy microgreens. Taken together, these data suggest that stomatal movement is specific for degree of microgravity simulation and that *Salmonella* may exploit altered stomatal physiology of plants subjected to simulated microgravity to ingress the apoplast to a greater extent than unrotated plants. Our finding of open stomatal apertures under rotation aligns with a previous study rotating the C3-photosynthetic monocot *Chlorophytum comosum* using a 3-D clinostat^28^. The authors observed stomatal apertures remained open after 24 h of gravistimulation under both light and dark conditions, while normal gravity control plants exhibited normal stomatal photo-regulation with closed apertures in the dark and open in the light. Elevated levels of the plant hormone auxin (indole-3-acetic acid) were observed in the shoots of gravistimulated plants offering a potential mechanistic explanation for the dramatic alterations to stomatal physiology under rotation which overrode light/dark photo-regulation^28^. Modified transport of auxin has been demonstrated to be an important driver of aboveground phenotypic alterations under microgravity^29,30^. Accumulation of auxin in shoot leaf tissues has previously been shown to maintain open stomatal apertures^31,32^. Our findings suggest enhanced plant susceptibility to bacterial pathogens under microgravity conditions, raising concerns regarding spaceflight food safety. Impaired transport of auxin in foliar and shoot tissues under gravistimulation may alter stomatal dynamics providing an advantage to bacterial pathogens for ingression. Altered stomatal physiology under microgravity may also have broader implications beyond food safety pertaining to photosynthetic efficiency, CO2 uptake, and phytoremediation aboard spacecraft.

Having shown that plants subjected to simulated microgravity were more susceptible to suppression of stomatal defense by *Salmonella*, we next tested for a correlation between gravistimulation and ingression depth (µm) of *Salmonella* in the foliar apoplast. Lettuce leaves were inoculated GFP-labeled *Salmonella* and vertically rotated at 4 RPM in the 2D clinostat or kept vertically positioned as a control, for 3, 6, and 9 h respectively. Following rotation, leaf tissue was fixed and stained for acquisition of Z-stacks by confocal microcopy to create a 3D reconstruction of the leaf apoplast. Plants rotated at 4 RPM displayed significantly deeper ingression and more colony forming units by *Salmonella* compared to unrotated pathogen-treated plants (Extended Data Figs. 2, 3, Extended Data Movies 2, 3). This data indicates that wider stomatal apertures under simulated microgravity allow for more effective bacterial ingression compared to unrotated plants. This also suggests modified phyllosphere and foliar endophyte community structures under microgravity as ingression dynamics appear altered. Taken together, our findings show that simulated microgravity may cause a detrimental above-ground physiological change in plants, creating an advantage for apoplastic colonization by *Salmonella*. We previously demonstrated that *Salmonella typhimurium* required a regulatory gene of *Salmonella* Pathogenicity Island 1 and a T3SS-2 translocon subunit to override stomatal defense in lettuce for apoplastic ingression^6^. The T3SS also played a critical role in persistence of *S. typhimurium* in planta^6^. The foliar application of *S. typhimurium* produced downregulation of the ABA biosynthetic gene LsNCED3 in roots, coinciding with suppression of stomatal closure^6^. While modulation of stomata in lettuce appears T3SS dependent, the mechanism of action remains unknown.

Having shown that *Salmonella* ingress stomatal apertures of plants under simulated microgravity to a seemingly greater degree compared to unrotated plants in terms of ingression depth and in planta estimated bacterial populations, we tested the ability of the PGPR *Bacillus subtilis* strain UD1022 to restrict stomatal apertures of lettuce under simulated microgravity, with and without foliar application of *Salmonella*. We inoculated lettuce roots with UD1022, allowing 48 h for root colonization, and followed with either a *Salmonella* foliar-application or mock treatment immediately prior to simulated microgravity at 4 RPM or an unrotated vertical control. We hypothesized that under simulated microgravity, UD1022 alone would restrict stomatal apertures and would counteract the suppression stomatal closure by *Salmonella* in a co-inoculation.

**Figure 3:**
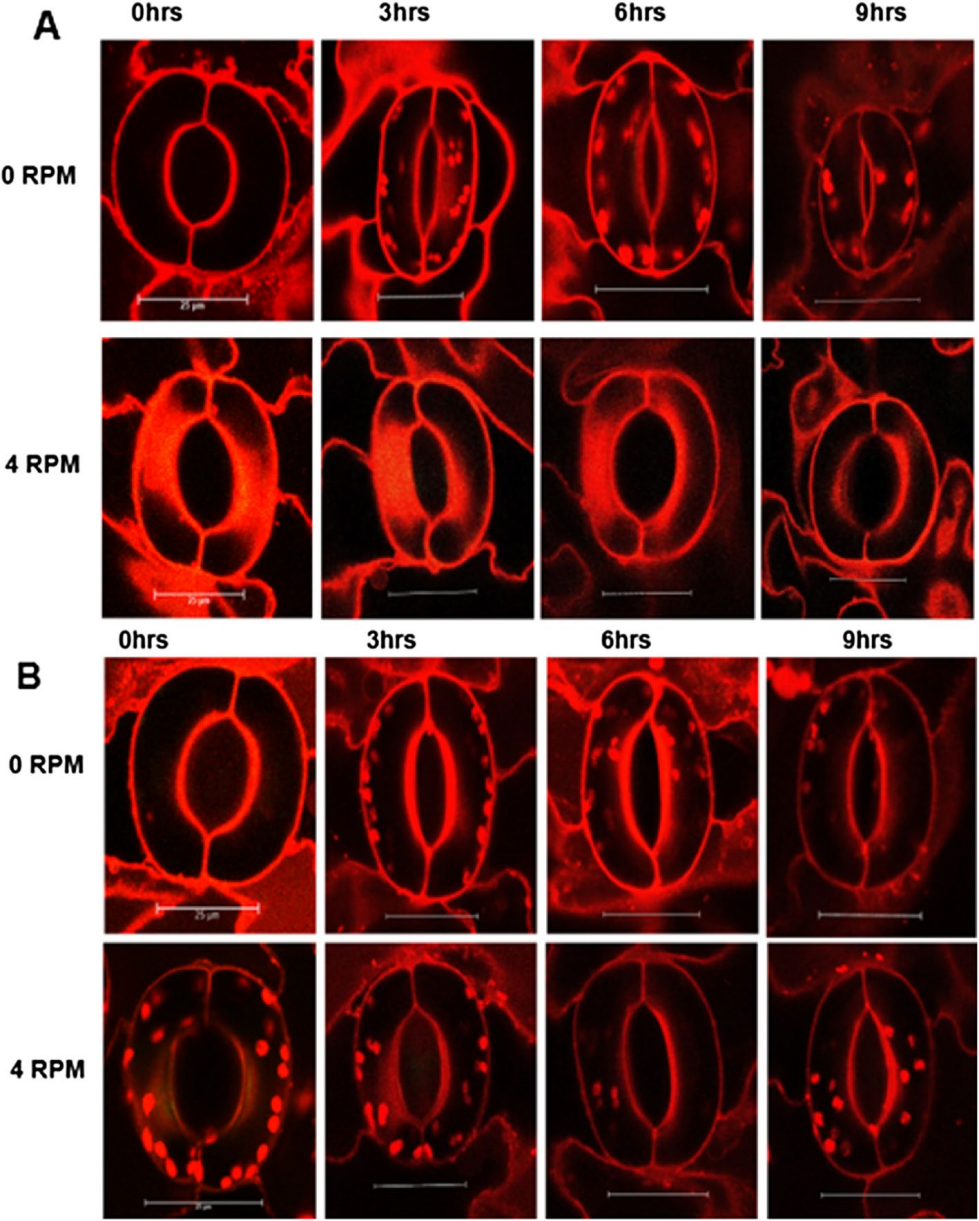
(**A**) Representative images of stomatal apertures overtime in lettuce plants with *B. subtilis* UD1022 root inoculation 4 RPM, or a 0 RPM vertical control. (**B**) Representative images of stomatal apertures overtime in lettuce plants with foliar application of *Salmonella* and UD1022 root inoculation. Average stomatal apertures were micro-graphed and aperture size was estimated from n = 75 stomates per timepoint.

**Figure 4:**
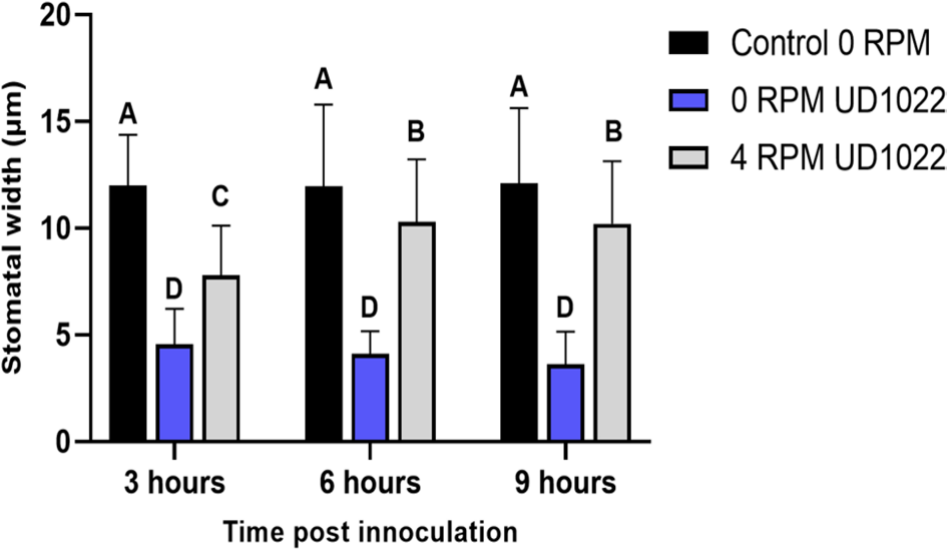
Average stomatal aperture width in lettuce plants with simulated microgravity following root treatment with *Bacillus subtilis* UD1022. Plants were rotated at 4 RPM ± UD1022 or maintained vertically as a control (0 RPM) ± UD1022. Stomatal apertures were imaged with confocal microscopy and aperture size was measured using ImageJ. Letters indicate statistically significant differences (p < 0.05) between treatments. n = 75 stomates per timepoint.

**Figure 5:**
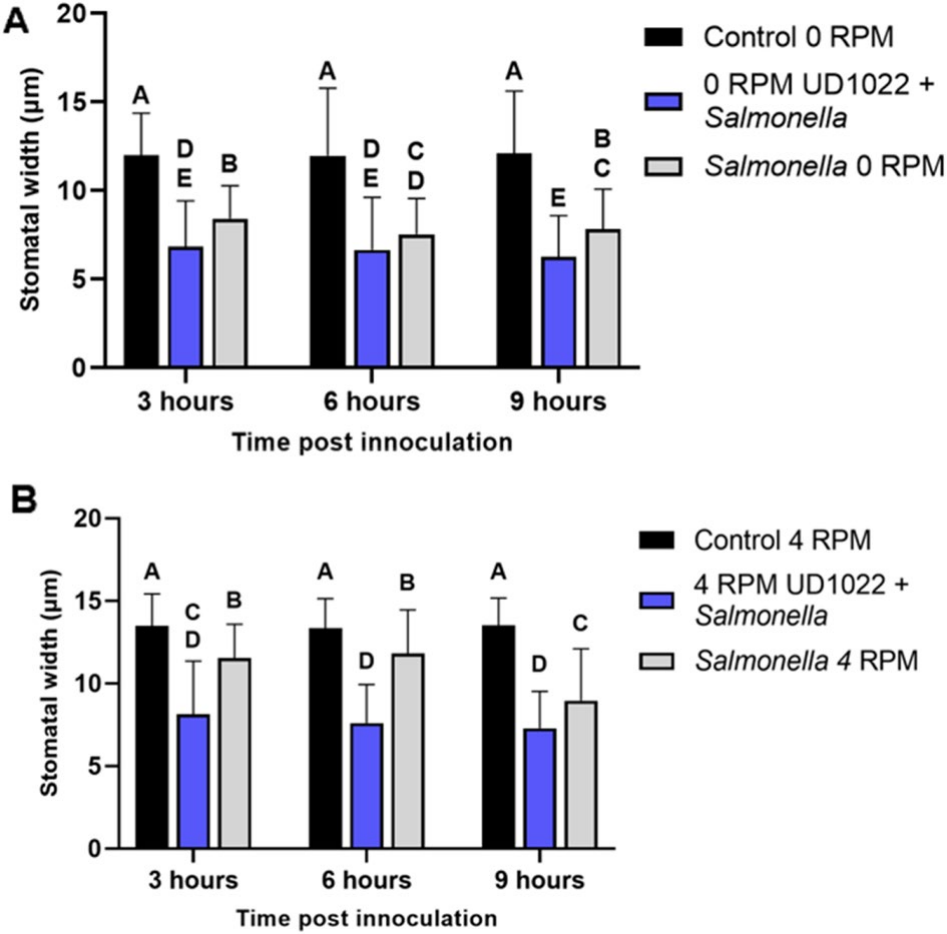
Stomatal apertures in lettuce plants with simulated microgravity following co-inoculation with UD1022 and *Salmonella* (**A**) maintained vertically as a control (0 RPM) (**B**) or rotated at 4 RPM. Plants were inoculated with root-treatment of UD0122 and foliar treatment of GFP-labeled *Salmonella enterica*. Unrotated plants treated with the co-inoculation of *S. enterica* + UD1022 *or S. enterica* alone were also imaged for stomatal apertures. Stomatal apertures were imaged with confocal microscopy and aperture size was measured using ImageJ. Letters indicate statistically significant differences (p < 0.05) between treatments. n = 75 stomates per treatment.

Surprisingly, plants rotated at 4 RPM with UD1022 alone exhibited significantly increased stomatal apertures compared to unrotated UD1022-treated plants, suggesting simulated microgravity inhibits stomatal closure by UD1022 (Figs. 3, 4). Unrotated control plants co-inoculated with UD1022 and *Salmonella* displayed reduced aperture sizes compared to *Salmonella* treatment alone, consistent with previous findings (Fig. 5A,B). Interestingly, co-inoculation under 4 RPM exhibited significantly wider stomatal apertures compared to co-inoculated plants under 4 RPM. This data suggests that physiological impact of simulated microgravity negates the efficacy of a commensal biocontrol known to restrict stomatal apertures and supports plant ingression by *Salmonella*. In accordance, a previous study found the biocontrol properties of *Pseudochrobactrum kiredjianiae* A4 against the fungal pathogen *Fusarium graminearum* on wheat seedlings to be diminished under simulated microgravity using a random positioning machine^33^.

UD1022 root application upregulates an ABA/Salicylic acid-dependent pathway to restrict stomata in *Arabidopsis* and lettuce^6,26^, and increases foliar ABA concentrations in *Arabidopsis*^26^. Inversely, the inability of UD1022 to restrict stomata under simulated microgravity may relate to temporal translocation of secondary messengers such as ROS, Ca^2+^ or mobile peptides that play a critical role in closing stomata under abiotic stress^34^. Identifying the key xylem-mobile elements involved in inversing stomatal closure by UD1022 under simulated microgravity will further elucidate the activity of biocontrols in spaceflight against plant ingression by bacterial foodborne pathogens. In the future, a variety of plant, biocontrol, and pathogen systems should be tested under both genuine and simulated microgravity conditions to illuminate this previously unexplored frontier of food safety.

## Conclusions

In summary, our tripartite system of lettuce, *Salmonella*, and *B. subtilis* demonstrates the first example of a biocontrol-pathogen interaction modulating above-ground physiology in plants under simulated microgravity. The unexpected finding that simulated microgravity negates the classic foliar stomatal restriction response by PGPR root-application opens new questions about how plant–microbe interactions may vary under microgravity conditions compared to normal gravity. Additionally, the inability of *B. subtilis* UD1022 to restrict stomata in plants subjected to simulated microgravity inoculated with *Salmonella* challenges the tripartite biocontrol model established under normal gravity conditions. These results are both intriguing and challenging as they indicate that human pathogens like *Salmonella* may exploit altered plant innate immunity under microgravity to contaminate plants more effectively in spaceflight conditions.

## Methods

### Plant culture

Seeds of *Lactuca sativa* L. var. Black Seeded Simpson (Johnny’s Seed Supply) were stored long-term (~ 1 month) in the dark at 4 °C inside their original seed packet. The collection and maintenance of *L. sativa* plants (cultivated), complies with the University of Delaware, national, and international guidelines, and legislation. Seeds were submerged in 25 mL, 50% germicidal bleach and deionized water and lightly shaken for 9 min inside a 50 mL conical tube. Bleach solution was removed, and seeds were rinsed trice for 30 s in 25 mL sterile DI water. 25 mL of 0.8% Phytagel media supplemented with 1% w/v sucrose and ½ strength Murashige and Skoog was poured in 90 × 90 cm square Petri dishes were allowed solidify in an aseptic laminar flow hood. Petri dishes were positioned vertically, and the top 30 cm of solidified media was removed using a sterilized X-ACTO knife to allow space for upward vertical growth of leaves and to create a flat surface of solid media for seeds to germinate upon with dishes in an upright position. Three sterilized seeds were placed two grid squares apart (26 mm) inside Petri dishes using sterile forceps and dishes were sealed using micropore tape. Square petri dishes were positioned vertically for 7 days at 25 °C under 250 micromoles per square meter per second (μmol m^−2^/s) at 25% relative humidity with a 12-h photoperiod. Following 7 days of growth, petri dishes were vertically positioned within the clinostat under 250 μmol m^−2^/s full spectrum LED lights (Shenzhen Houyi Network Technology Co., Ltd) 1 h prior to bacterial treatment or rotation. Each square Petri dish contained n = 3 plants. For each treatment experimental timepoint (0, 3, 6, 9 h post ± rotation/± inoculation), one Petri dish was sampled, with one leaf sampled from each plant (n = 3 leaves per timepoint).

### Bacterial culture

*Salmonella enterica* subsp. *enterica* serovar Typhimurium GFP 14028GFP™ supplied by the American Type Culture Collection was stored in 40% glycerol in − 80 °C freezer for long-term storage. Before experimental use, bacteria were streaked from a glycerol stock on LB (Miller Broth) with 0.8% agar and 100 μg/mL ampicillin and incubated at 37 °C for 24 h. Following solid media incubation, an individual single colony forming unit (CFU) was moved using a sterile loop into a 50 mL conical tube containing 25 mL LB (Miller Broth) with 100 μg/mL ampicillin. Liquid cultures were incubated at 250 RPM at 37 °C for 24 h. Following liquid media incubation, a bacterial pellet was obtained by centrifugation at 4000 RPM for 5 min. The pellet was resuspended by vortexing and washed twice in 25 mL phosphate buffered saline (PBS) at a pH of 7.4, followed by a final suspension in PBS. The optical density (OD) was measured at 600 nm with a Bio-Rad SmartSpec + spectrophotometer (Bio-Rad Inc.) and adjusted to an OD of 0.8 with a final concentration of 10^8^ cells/mL. All experiments involving *S. enterica* Typhimurium used the methods described above and experiments were immediately conducted following final adjustment of OD.

*Bacillus subtilis* UD1022 was stored in 40% glycerol in − 80 °C freezer for long-term storage. Before experimental use, UD1022 was streaked on LB (Miller Broth) with 0.8% agar and incubated at 30 °C for 24 h. Following solid media incubation, an individual single colony forming unit (CFU) was moved using a sterile loop into a 50 mL conical tube containing 25 mL LB (Miller Broth). Liquid cultures were incubated at 250 RPM at 37 °C for 24 h. Following liquid media incubation, a bacterial pellet was obtained by centrifugation at 4000 RPM for 5 min. The pellet was resuspended by vortexing and washed twice in 25 mL phosphate buffered saline (PBS) at a pH of 7.4, followed by a final suspension in PBS. The optical density (OD) was measured at 600 nm with a Bio-Rad SmartSpec + spectrophotometer (Bio-Rad Inc.) and adjusted to an OD of 0.02 with a final concentration of 10^6^ cells/mL.

### Root application of *Bacillus subtilis* UD1022

Following OD adjustment, the bacterial culture was immediately used for lettuce root colonization. The bacterial culture was vortexed for 5 s and 5 μL of culture was pipetted onto the lateral roots and root tip of each of the three 5-day old plants contained within a square Petri dish. Roots were present along the surface of the Phytagel substrate allowing for bacterial colonization. Plants were maintained for 48 h at 25 °C under 250 μmol m^−2^/s at 25% relative humidity with a 12-h photoperiod to allow for root colonization. Following root colonization, 7-day old plants were used in subsequent stomatal width assays.

### Foliar application of *Salmonella*

A horsehair brush was sterilized by submergence in 100% ethanol for 60 s, followed by three vigorous 30 s washes in sterile DI water, to inoculate the abaxial side of leaves with *Salmonella enterica* Typhimurium. Freshly prepared bacterial culture was vortexed, and the hair end of brush was submerged in the culture for 5 s. Abaxial leaf surfaces lightly brushed a single time inside an aseptic laminar flow hood. Every available leaf was brushed once on its abaxial surface, with 7-day old plants reliably yielding four true leaves. The procedure described above was used for inoculation of all three plants contained in a single square Petri dish. Prior to inoculation of plants in a subsequent Petri dish, sterilization of the brush with ethanol was repeated and the brush was submerged again in the bacterial culture following vortexing. Uninoculated control plants were brushed on the abaxial side of their leaves with sterile DI water using a horsehair brush sterilized with ethanol as described above. For UD1022 co-inoculation experiments foliar application of *Salmonella*/mock treatment proceeded 48 h after root inoculation with UD1022.

### Clinostat prototype

A two dimensional (2-D) slow-rotating clinostat was kindly provided by Chandran R. Sabanayagam of the Delaware Biotechnology Institute (15 Innovation Way, Newark, DE, USA 19711) (see Extended Data Fig. 4, Extended Data Movie 4). Two full-spectrum LED strips (Shenzhen Houyi Network Technology Co., Ltd) were installed parallel with each other inside the clinostat so that they continuously rotate alongside plants. LED light strips were programmed to a light intensity of 250 μmol m^−2^/s. An adjustable plexiglass rack was installed inside the clinostat and raised to 40 mm so that the surface of the Phytagel media within the square Petri dishes aligned with the central axis of rotation.

### Clinostat gravistimulation

Four square Petri dishes containing three plants each (n = 12) were vertically secured with tape to the surface of the Plexiglass rack facing the LED lights. Plants were vertically rotated at 2 or 4 RPM with the longitudinal root to shoot axis perpendicular to the rotation plane. Rotation was immediately initiated following inoculation of plants with bacteria. Plants were immediately sampled at experimental timepoints of 0-, 3-, 6-, and 9-h following rotation. An unrotated control (0 RPM) was left in the upright vertical position for all experimental timepoints.

### Stomatal width assay

For each experimental time point (0, 3, 6, 9 h) a single square Petri dish was removed from the clinostat following rotation. A single leaf was sampled from each of the 3 plants contained within the dish (n = 3 leaves per timepoint). Leaves are immediately excised from petioles using a sterile X-ACTO knife and moved with sterile forceps to 10 mL beakers containing a fixative solution of 4% paraformaldehyde and PBS. Leaves were weighed down with glass blocks to prevent them from floating on the surface of the fixative solution. The leaves were left submerged in the fixative solution for 24 h at room temperature (25 ± 3 °C). The leaves were then lightly rinsed in DI water and floated abaxial side down in 1 mg/mL propidium iodide in DI water for staining. Following staining, leaves were lightly rinsed in DI water and mounted abaxial side down in separate glass bottomed Nunc Chambers with 200 μL DI water and sterile glass blocks placed on top. Leaves were imaged with inverted-confocal laser microscopy using a Leica STELLARIS 8 tauSTED located in the University of Delaware Bioimaging Core. Stomata were randomly measured from the abaxial side of each of the 3 leaves sampled per timepoints (n = 25 stomata per leaf). Experiments were conducted in duplicates (n = 150 stomata total per timepoint). Images were captured using an 86× water immersion magnification objective + 1.28 × digital zoom and a numerical aperture of 1.20. Images were acquired at 2176 × 2176 pixels per frame, pixel size of 0.049 μm, bit-depth of 12, Airy Units set to 1.0, a unidirectional X axis scan speed of 400 Hz, pixel dwell time of 1.35 µs, frame rate of 0.331/s, and a physical length of 106.02 × 106.02 μm per frame. Propidium iodide stain was excited with a 489 nm laser at 2% intensity through a 488/561 bandpass filter with the emission spectra set to 509–562 nm and digital gain set to 20%. The GFPmut3 protein was excited with a 504 nm laser at 7% intensity through a 360/40 bandpass filter with emission spectra set to 588–725 nm and digital gain set to 2.5%. Following imaging, stomatal apertures were measured using ImageJ. The ellipse tool was used to trace the stomatal aperture and the minor axis was calculated to provide a precise measurement of width.

### Ingression analysis

Immediately following foliar inoculation of bacteria, plants were rotated or maintained in a vertical position (control) for experimental timepoints of 3, 6, and 9 h. For each experimental time point (0, 3, 6, 9 h) a single square Petri dish was removed from the clinostat following rotation. A single leaf was sampled from each of the 3 plants contained within the dish (n = 3 leaves), and 25 stomata were randomly measured per leaf (n = 75 stomata per timepoint) for depth of bacterial ingression. Leaves are immediately excised from petioles using a sterile X-ACTO knife and moved with sterile forceps to 10 mL beakers containing a fixative solution of 4% paraformaldehyde and PBS. Leaves were weighed down with glass blocks to prevent them from floating on the surface of the fixative solution. Leaves were left submerged in the fixative solution for 24 h at room temperature (25 ± 3 °C). The leaves were then lightly rinsed in DI water and stained in CellMask Orange plasma membrane stain for 10 min. Following another light rinse in DI water, leaves were mounted abaxial side down in separate glass bottomed Nunc Chambers with 200 μL DI water and sterile glass blocks placed on top. A single Z stack was acquired for each leaf from the leaf surface to the deepest visible GFP-tagged bacteria within the apoplast using a Andor Dragonfly 600 Spinning Disc Confocal microscope located in the University of Delaware Bioimaging Core. CellMask Orange was excited using a 561 nm laser with emission captured at 650 nm, GFPMut3 protein was excited with 488 nm with emission captured at 521 nm, and chlorophyll was excited at 638 nm with emission captured at 685 nm. Z stacks were imaged at 16 bits with a voxel size of X 0.151, Y 0.151, Z 0.308. Depth of bacterial ingression was calculated in Imaris Microscopy Image Analysis Software by Oxford Instruments. The straight vertical distance (μm) between the Z plane at the surface of stomatal guard cells and the Z plane containing the deepest visible bacterium was recorded.

### CFU enumeration

A single leaf was sampled from each plant in 4 square Petri dishes (n = 12) at 9 h following bacterial inoculation of leaves and rotation at 4 RPM or at 9 h following bacterial inoculation of leaves and no rotation. Leaves were immediately excised from petioles with sterile X-ACTO knife at experimental time points and weighed for fresh weight. The leaves were then sterilized in 100% ethanol for 5 s to eliminate all bacteria on the leaf surface which have internalized within stomata. The leaves were lightly rinsed in sterile DI water and each leaf was placed in a separate 2 mL Eppendorf tube. 1 mL of sterile PBS was added to each tube and the leaf was thoroughly ground using a sterile plastic pestle and lightly vortexed to yield a homogenous mixture. The homogenized mixture was serially diluted at a 1:10 ratio by removing 100 μL and adding to an Eppendorf tube containing 900 μL of sterile PBS for a 10^–1^ dilution. This was repeated to reach a 10^–3^ dilution. Each dilution tube was vortexed and three replicates of 10 μL were removed and spread across separate 120 mm diameter circular Petri dishes containing LB media (Miller Broth) with 0.8% agar. Petri dishes were sealed with parafilm and incubated for 12 h at 37 °C. Single colony forming units (CFUs) were counted and CFUs per gram of fresh weight leaf tissue were calculated.

### Statistical analysis

Data were analyzed using a one-way ANOVA to determine the effect of treatments, and a Tukey–Kramer HSD was used to compare means of stomatal width and bacterial ingression depth over time by using JMP software (JMP v.14; SAS Institute Inc., Cary, NC) at a significance level of p < 0.05.

### Supplementary Information

Supplementary Legends.

Supplementary Movie 1.

Supplementary Movie 2.

Supplementary Movie 3.

Supplementary Movie 4.

Supplementary Figures.

## Data Availability

All data supporting the findings of this study are available within the paper, its Extended Data and Supplementary Information.
