# Supplementary material for: Simulated microgravity facilitates stomatal ingression by Salmonella in lettuce and suppresses a biocontrol agent
Source: Sci Rep. 2024 Jan 9;14:898. doi: 10.1038/s41598-024-51573-y (PMC10776768; doi:10.1038/s41598-024-51573-y)
Supplement: Supplementary file 1 — Supplementary Legends. [file 41598_2024_51573_MOESM1_ESM.docx]

**Legends to Extended data.**

**Extended data Figure 1:** Negative gravitropic response in lettuce roots subjected to simulated microgravity for 7 days at 4 RPM. The arrows indicate roots of lettuce plants skewed horizontally to perceive the change gravitational plane.

**Extended data Figure 2:** Stomatal depth analysis in lettuce plants with simulated microgravity in the presence of *Salmonella* at 4 RPM or 0 RPM vertical control. Plants were inoculated with GFP-labeled *Salmonella enterica*. Depth of bacterial ingression was measured by analysis of Z stacks with Imaris Microscopy Image Analysis Software. n=75 stomates per treatment. Letters indicate statistically significant differences (p < 0.05) between treatments.

**Extended data Figure 3:** Individual colony forming units (CFUs) of *Salmonella* isolated from single leaves (n=5) of plants exposed to 0 RPM control or 4 RPM for 9 hours following bacterial inoculation. Each sampled leaf was taken from a distinct plant. Significance between treatments was determined with a t-test and is denoted in the figure legend by *.

**Extended data Figure 4:** 2-D clinostat used in the study for simulated microgravity.

**Legends to Extended data movies.**

**Movie#01:** Time lapse showing the entry of *Salmonella* (in green) in lettuce via stomate. Time lapse was created using ZEISS Celldiscoverer 7. 472

**Movie#02:** 3D representation of ingression by *Salmonella* (in red) in lettuce apoplast via stomates. Video created using Leica STELLARIS 8 tauSTED.

**Movie#03:** Individual stomate with Salmonella showing attachment of Salmonella at the guard cell level. Video created using Leica STELLARIS 8 tauSTED.

**Movie#04:** The functional prototype showing rotations with the lettuce plants.
