# Supplementary material for: Simulated microgravity facilitates stomatal ingression by Salmonella in lettuce and suppresses a biocontrol agent
Source: Sci Rep. 2024 Jan 9;14:898. doi: 10.1038/s41598-024-51573-y (PMC10776768; doi:10.1038/s41598-024-51573-y)
Supplement: Supplementary file 6 — Supplementary Figures. [file 41598_2024_51573_MOESM6_ESM.pptx]

## Slide 1
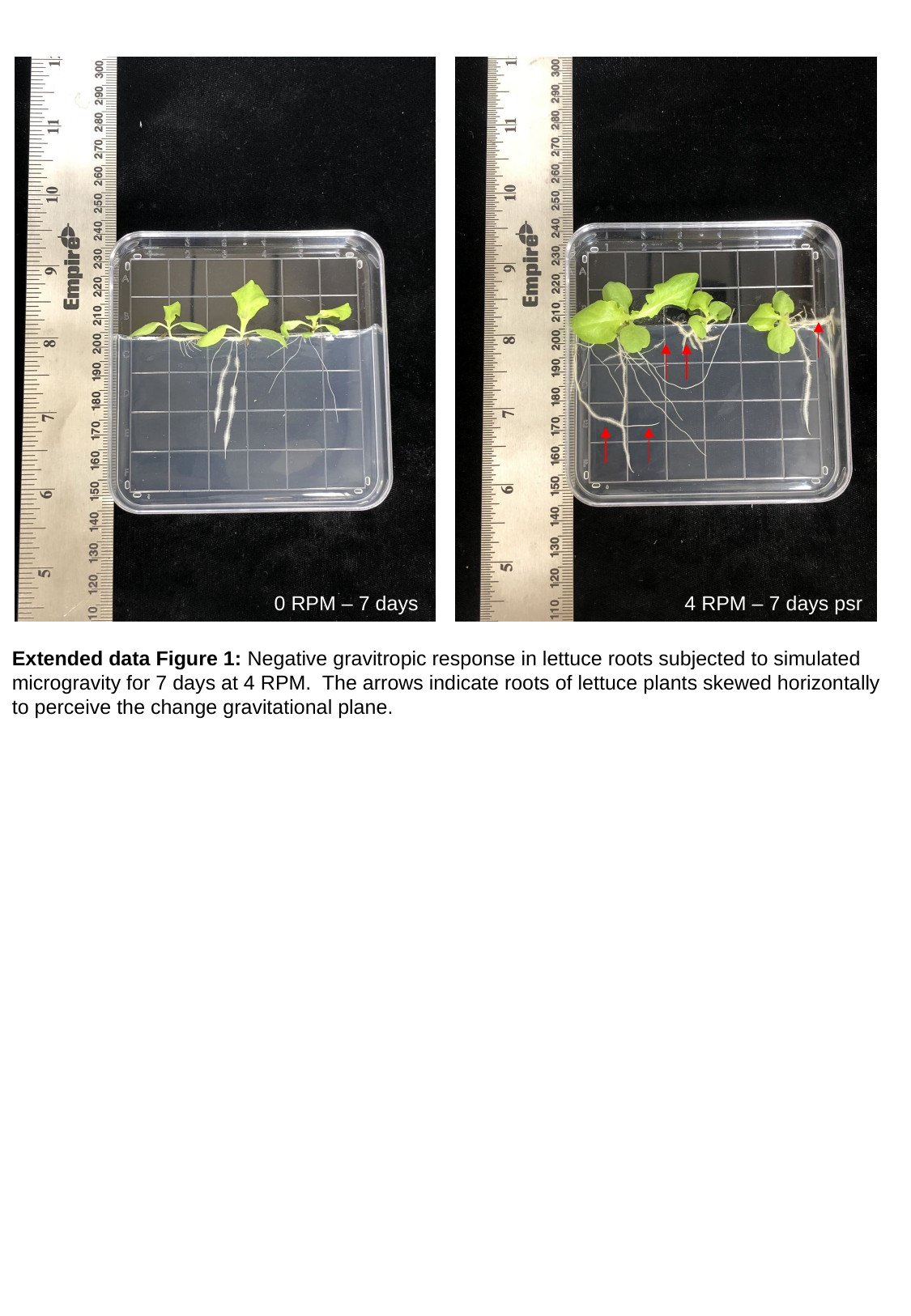

0 RPM – 7 days
4 RPM – 7 days psr
Extended data Figure 1: Negative gravitropic response in lettuce roots subjected to simulated microgravity for 7 days at 4 RPM. The arrows indicate roots of lettuce plants skewed horizontally to perceive the change gravitational plane.

## Slide 2
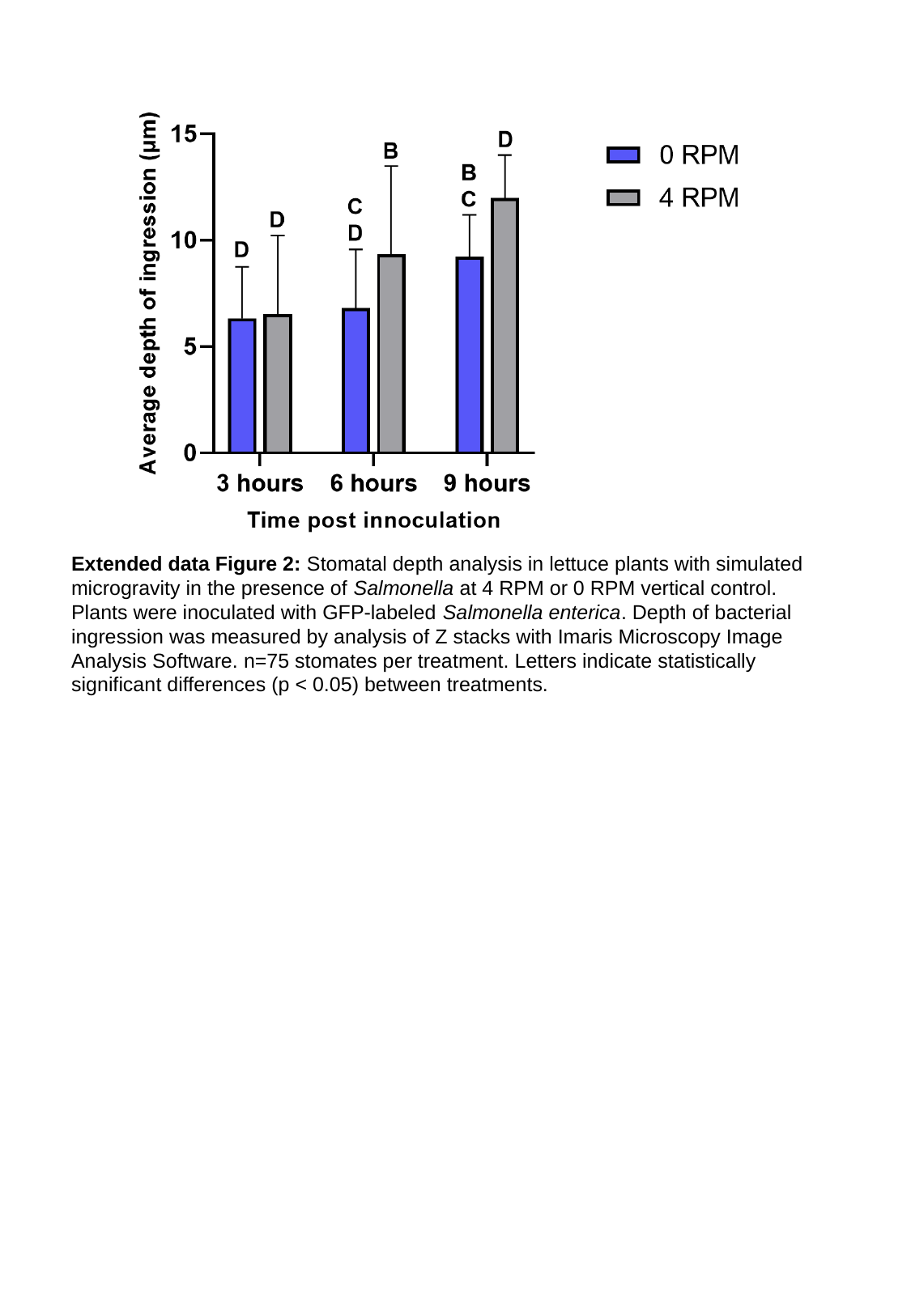

Extended data Figure 2: Stomatal depth analysis in lettuce plants with simulated microgravity in the presence of Salmonella at 4 RPM or 0 RPM vertical control. Plants were inoculated with GFP-labeled Salmonella enterica. Depth of bacterial ingression was measured by analysis of Z stacks with Imaris Microscopy Image Analysis Software. n=75 stomates per treatment. Letters indicate statistically significant differences (p < 0.05) between treatments.

## Slide 3
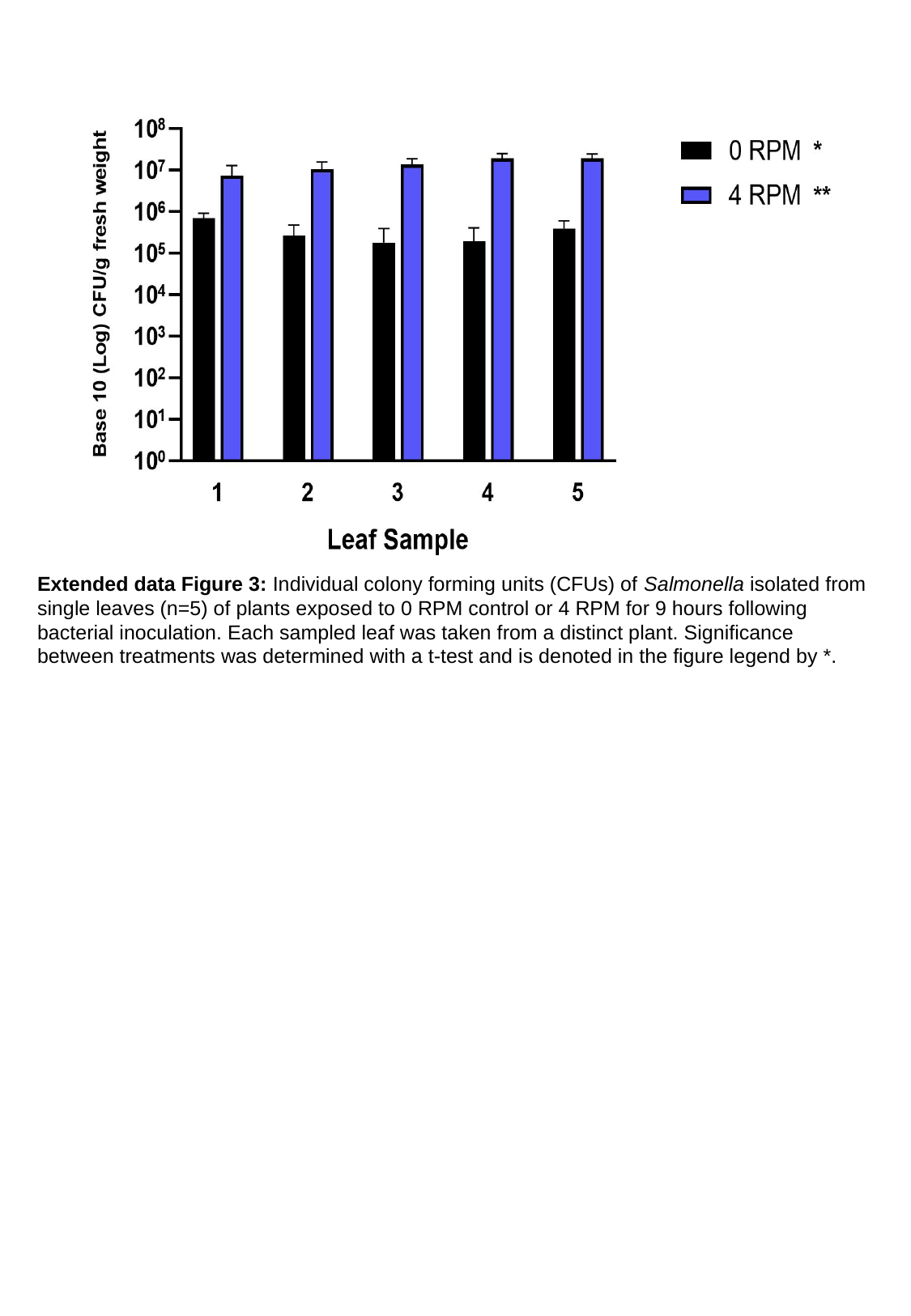

Extended data Figure 3: Individual colony forming units (CFUs) of Salmonella isolated from single leaves (n=5) of plants exposed to 0 RPM control or 4 RPM for 9 hours following bacterial inoculation. Each sampled leaf was taken from a distinct plant. Significance between treatments was determined with a t-test and is denoted in the figure legend by *.

## Slide 4
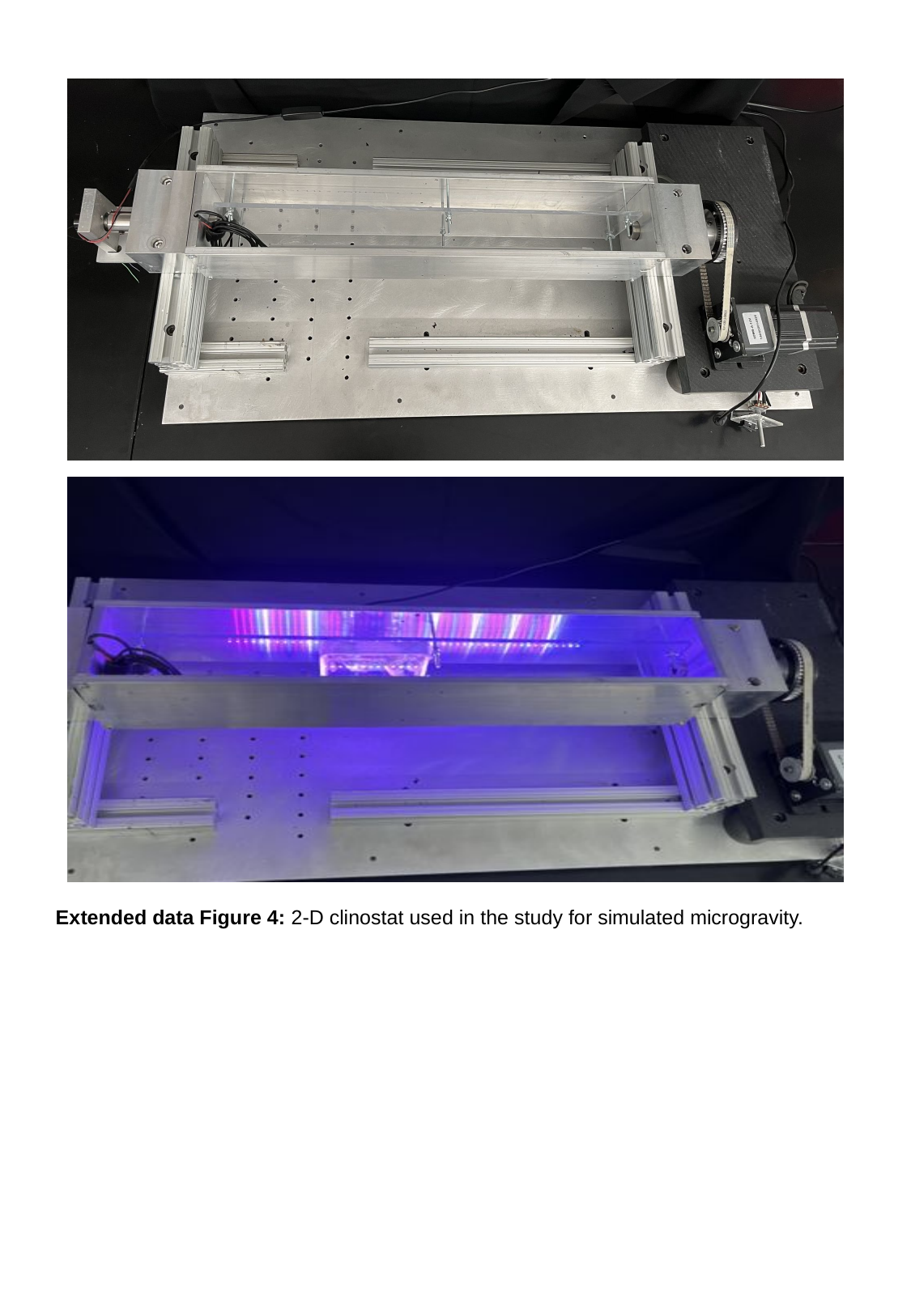

Extended data Figure 4: 2-D clinostat used in the study for simulated microgravity.
